# Beyond the Trial: Systematic Review of Real-World Uptake and Engagement With Digital Self-Help Interventions for Depression, Low Mood, or Anxiety

**DOI:** 10.2196/jmir.9275

**Published:** 2018-06-06

**Authors:** Theresa Fleming, Lynda Bavin, Mathijs Lucassen, Karolina Stasiak, Sarah Hopkins, Sally Merry

**Affiliations:** ^1^ Faculty of Health Victoria University of Wellington Wellington New Zealand; ^2^ Department of Psychological Medicine The University of Auckland Auckland New Zealand; ^3^ School of Health, Wellbeing and Social Care The Open University Milton Keynes United Kingdom

**Keywords:** e-therapy, mobile applications, eHealth, depression, anxiety

## Abstract

**Background:**

Digital self-help interventions (including online or computerized programs and apps) for common mental health issues have been shown to be appealing, engaging, and efficacious in randomized controlled trials. They show potential for improving access to therapy and improving population mental health. However, their use in the real world, ie, as implemented (disseminated) outside of research settings, may differ from that reported in trials, and implementation data are seldom reported.

**Objective:**

This study aimed to review peer-reviewed articles reporting user uptake and/or ongoing use, retention, or completion data (hereafter *usage data* or, for brevity, *engagement*) from implemented pure self-help (unguided) digital interventions for depression, anxiety, or the enhancement of mood.

**Methods:**

We conducted a systematic search of the Scopus, Embase, MEDLINE, and PsychINFO databases for studies reporting user uptake and/or usage data from implemented digital self-help interventions for the treatment or prevention of depression or anxiety, or the enhancement of mood, from 2002 to 2017. Additionally, we screened the reference lists of included articles, citations of these articles, and the titles of articles published in *Internet Interventions*, *Journal of Medical Internet Research (JMIR)*, and *JMIR Mental Health* since their inception. We extracted data indicating the number of registrations or downloads and usage of interventions.

**Results:**

After the removal of duplicates, 970 papers were identified, of which 10 met the inclusion criteria. Hand searching identified 1 additional article. The included articles reported on 7 publicly available interventions. There was little consistency in the measures reported. The number of registrants or downloads ranged widely, from 8 to over 40,000 per month. From 21% to 88% of users engaged in at least minimal use (eg, used the intervention at least once or completed one module or assessment), whereas 7-42% engaged in moderate use (completing between 40% and 60% of modular fixed-length programs or continuing to use apps after 4 weeks). Indications of completion or sustained use (completion of all modules or the last assessment or continuing to use apps after six weeks or more) varied from 0.5% to 28.6%.

**Conclusions:**

Available data suggest that uptake and engagement vary widely among the handful of implemented digital self-help apps and programs that have reported this, and that usage may vary from that reported in trials. Implementation data should be routinely gathered and reported to facilitate improved uptake and engagement, arguably among the major challenges in digital health.

## Introduction

### Background

Digital interventions (including online or computerized programs or apps) have been shown to be efficacious for depression and anxiety [1-4] and they provide the opportunity to extend psychological therapy to people who might otherwise not receive it [3-5]. Adherence to digital interventions is important for therapeutic gains [3,6] and is generally satisfactory relative to face-to-face interventions [7]. Self-help interventions, provided without guidance or personal support, might reach users who are unable or unwilling to seek help and may be scaled up at lower cost than interventions involving assistance [4,5]. Adherence to self-help is generally lower than that to guided interventions [8], although approaches such as persuasive design and telepresence may enhance retention [5,9] and advantages such as scalability mean that self-help remains worthy of attention.

Interventions may have poorer outcomes when implemented in community or clinical settings than they do in research trials [10-12]. Proven interventions can fail in the real world because translation from research trials may involve changes in the conditions under which the original results were obtained [10,11]. For instance, trials may exclude participants with complex issues, and trial participants may have additional motivations to complete interventions, such as to please researchers or to help others. Trial participants might also benefit from assessment effects or face-to-face contacts that are independent of the intervention [10]. Digital technology is evolving rapidly; hence, digital interventions that are not updated or refined following trials are at risk of becoming dated and, consequently, less appealing by the time they are available outside of research settings [13]. For these reasons, it is important to examine the use of digital interventions in real-world settings. Examining self-help interventions in isolation provides the opportunity to highlight differences between individual interventions of this type.

### Objectives

In this study, we aimed to systematically review peer-reviewed articles reporting user uptake (eg, number of users, registrations, or downloads) and/or ongoing use, adherence, retention, or completion data (hereafter *usage data* or, for brevity, *engagement*) from implemented digital self-help interventions for the prevention or treatment of anxiety or depression, or for the enhancement of mood. We note that aspects of engagement other than usage data (eg, emotional involvement) are important [14-16]. However usage data are widely reported, are important for efficacy [6,7], and are the focus here. We identified no prior systematic reviews on this topic.

## Methods

### Search Strategy

Electronic searches were conducted of the Scopus, Embase, MEDLINE, and PsychINFO databases. The following search terms were used in Scopus, and the equivalent search was repeated on the Ovid Embase, MEDLINE, and PsychINFO databases:

( TITLE-ABS-KEY ( implementation OR “real world” OR real-world OR naturalistic OR observational OR “open access” OR public OR “publicly available” OR “publically available” OR deployment OR community OR nationwide OR national OR regist* OR dissemination ) AND TITLE-ABS-KEY ( computerized PRE/5 therap* ) OR etherap* OR e-therap* OR ( online PRE/5 intervention* ) OR ( online PRE/5 treatment* ) OR ( internet PRE/5 intervention* ) OR ( website PRE/5 intervention* ) OR ( web-based PRE/5 intervention* ) OR ( web-based PRE/5 treatment* ) OR “smartphone app*” OR “mobile app*” OR “smartphone intervention*” OR “smartphone program*” OR “mobile program*” OR “mobile intervention*” OR mhealth OR mtherapy ) AND TITLE-ABS-KEY ( depression OR anxiety OR mood OR “mental health” OR “psychological wellbeing” ) AND TITLE-ABS-KEY ( uptake OR adopt* OR regist* OR enrol* OR recruit* OR logon OR “logged on” OR usage OR adherence OR compliance OR complet* OR attrition OR “drop out” OR dropout OR drop-out ) ).

The search strategy was developed in partnership with a specialist research librarian. The search of the Ovid databases included “mp” (“multi-purpose”), thus incorporated all subject headings in which one or more word(s) matched the search term.

The following journals were hand-searched from their inception (all post 2002) up to and including their February 2017 issue: *Internet Interventions*, *Journal of Medical Internet Research (JMIR)*, and *JMIR Mental Health*. Finally, a hand search was conducted of the included studies’ reference lists, and the titles of articles that had cited the included papers.

### Inclusion and Exclusion Criteria

Articles were included in the review if they:

- were digital (computerized or online programs or apps) self-help/unguided interventions explicitly described as being for the prevention or treatment of depression or anxiety, or for the enhancement or improvement of mood;
- reported data on user uptake (eg, number of users, registrations, or downloads) and/or usage, adherence, or attrition (eg, number or percentage of users beginning, completing, or partially completing the intervention, or using the intervention for a specified period of time);
- reported implementation (dissemination/observational) data; and
- were published in the peer-reviewed literature between January 1, 2002, and March 8, 2017.

Articles were excluded if they:

- were pilot, exploratory, or feasibility studies; randomized controlled trials (RCTs); or protocol papers;
- were studies in which users were subject to assessments for research purposes, over and above what would normally be embedded in the intervention (ie, interventions could be included if routinely administered assessments were embedded as part of the self-help tool, but were excluded if users were subject to face-to-face or additional assessments for research purposes);
- reported findings from supported digital interventions (ie, supported by a therapist or where other human support was provided) or interventions that utilized a moderator or that were blended (eg, an adjunct to face-to-face therapy); and/or
- were not available in English.

### Study Selection

Two authors (TF and LB) independently screened all retrieved titles, and then read the abstracts of all potentially relevant articles. Articles identified by one or both screening author(s) as potentially relevant were reviewed in full text. For each article excluded at the full-text review, the main reason for exclusion was recorded.

### Data Extraction

The characteristics of all the included articles were coded by 2 of the 3 authors (LB, KS, and SH) and checked by 1 author (ML). The authors utilized a data extraction template that was developed for this systematic review and piloted on 2 of the full-text articles. Any discrepancies were resolved by referring to the original article and via discussion. The following characteristics and data were extracted:

- article reference details and data collection period;
- intervention characteristics: name of the intervention, intervention type (eg, online program, computerized program such as CD-ROM, smartphone app), condition treated, therapeutic modality, intervention length, features of gamification and navigation, and whether previously trialled and reported in the peer-reviewed literature;
- number of persons registering for or downloading the intervention;
- registration rate (the percentage of visitors to the intervention’s Website who then registered for the intervention), where data allowed;
- indicators of *at least minimal use*, such as number or percentage of users who began or used the intervention at least once or, where those data were not reported, number or percentage of users who completed at least one module or one assessment;
- indicators of *moderate use* (ie, more than *at least minimal use* but less than *completion or sustained use*), such as number or percentage of users completing a specified number or proportion of modules, or number of logins, or use for a specific period of time; and
- indicators of *completion or sustained use*, the number or percentage of users completing the intervention or, where no end-point was specified, the number or percentage using it for at least 6 weeks. Where neither of these were specified, the number or percentage of users who completed a final assessment or assessment at 6 weeks or more was recorded.

## Results

### Study Selection

**Figure 1:**
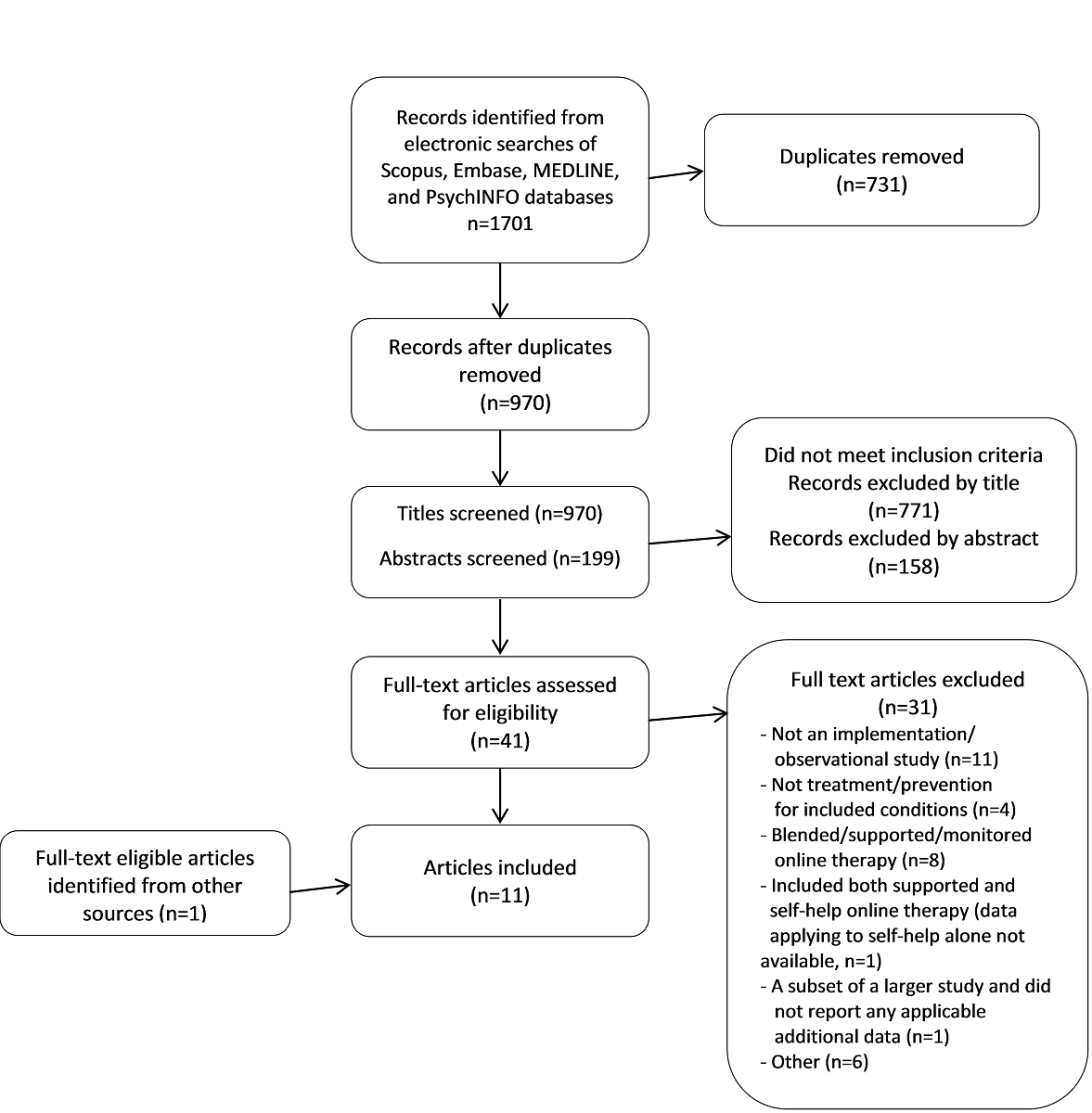
Flow diagram of article selection.

The database search yielded 1701 records, of which 970 remained after the removal of duplicates as shown in Figure 1. The initial title screening excluded 771 records, and the abstract screening excluded a further 158 records. The full texts of the remaining 41 articles were reviewed, of which 10 passed the inclusion and exclusion criteria. Several papers required detailed consideration. A paper by Al-Asadi and colleagues [17] was excluded because it was not possible to separate data for those persons who selected therapist-assisted self-help from those who selected pure self-help. Another paper by Al-Asadi and colleagues [18] was included because results for those receiving pure self-help were provided. A paper by Menzies and colleagues [19] was included, despite being described as a trial, because the intervention was available online without referral, no researcher contact was involved, no assessments beyond those routinely included in the intervention were used, and there was no randomization. An intervention for posttraumatic stress disorder (PTSD) [20] was included as PTSD was classified as an anxiety disorder in the Diagnostic and Statistical Manual, Fourth Edition, Text Revision (DSM-IV-TR) [21], during most of our review period (ie, 2002-2017), although it was reclassified in the fifth edition of the DSM, issued in 2013 [22]. The hand-searching process identified one additional article [23] (Happify) for inclusion in the systematic review, bringing the total of eligible articles to 11.

### Study and Program Characteristics

The 11 articles reported implementation data from 7 different interventions. Five articles reported on the original (ie, Mark I) or updated (ie, Mark II) version of the MoodGYM program [24-28], and the remaining 6 each described a unique intervention [18-20,23,29,30].

Study and program characteristics are summarized in Table 1. All of the included interventions were available without referral and were free to the user, apart from one that offered purchases or subscription for some content [23]. All were described as based on cognitive behavioral therapy (CBT) or utilizing CBT among other therapeutic modalities (eg, positive psychology), apart from one that did not specify the modality used [18]. Four interventions were online programs of fixed length, using sequential navigation (where content is provided in a specific order), or a choice of sequential or open navigation. The remaining 3 interventions were available via smartphone as an app [20], a suite of apps [30], or could be accessed as an app or online [23]. These app-based interventions (hereafter *apps*) had no fixed length and used open navigation. Notably, the articles reporting on apps were all relatively recent (ie, from 2015 to 2016).

The efficacy of one program (MoodGYM) has been empirically supported through RCTs [25,31,32]. For 3 interventions there was some evidence: a small, exploratory pre-post trial found beneficial effects for CBTPsych [33]; a single-arm trial, which included coaching, showed significant therapeutic improvements for users of Intellicare apps [30]; and, for PTSD Coach, there was both a pilot RCT reporting modest, nonsignificant effects [34] and a subsequent RCT reporting significant therapeutic effects at post-treatment [35]. At the time of writing, we found no published studies examining the efficacy of 3 interventions: Anxiety Online, Happify, and HDep.

### Uptake and Usage Data

User uptake (registrations or downloads) and usage data for each intervention are summarized in Table 2.

The number of registrations or downloads were reported in 8 papers. The remaining 3 studies reported related indicators as shown in Table 2. Registrations or downloads varied markedly from an average of 8 to at least 40,053 per month. Across the 7 interventions (and using the Mark II community user registration rate for the MoodGYM program), the median number of registrations or downloads was 401 per month, and the mean when excluding the lower and upper outliers was 2098 per month. There were 3 interventions with thousands of downloads or registrants per month, including 2 of the 3 apps, PTSD Coach, and Happify [20,23], and 1 of the 4 online programs, MoodGYM Mark II [27].

In Table 2, for MoodGYM, the number of assessments completed is likely to be similar to the number of modules completed, but not interchangeable. Assessments are completed at the beginning of a module and some users may not complete modules that they commence. Furthermore, for the Mark I version, assessments were not compulsory (a module could be completed without doing the assessment associated with that module); therefore, users may have skipped assessments.

Just 1 study, which was of MoodGYM Mark II [27], reported both the number of website visitors and the number of registrations, allowing the calculation of a registration rate, which was 42.2%.

Available measures indicating *at least minimal use* were limited and varied widely as shown in Table 2, making direct comparisons challenging. Within these limitations, CBTPsych [19] had the highest percentage of registrants engaging in at least minimal use, although given the low number of registrations per month, this was very few individuals.

Next, we reviewed indicators of moderate use. Again, there was little consistency in available data. Of the online programs: up to 16% of MoodGYM users completed 2 or more modules or assessments (embedded within the modules) out of 5 [24-28]; 10% of HDep users completed module 4 of this 7-module program (although they could miss individual modules) [29]; and 39% of CBTPsych users completed at least 4 of the 7 modules [19]. The apps or blended interventions reported quite different measures. Over 40% of PTSD Coach users continued to use it a month after installation [20]. For Happify, 20.6% of those who had completed an initial assessment also completed a noncompulsory assessment 2 weeks later, and 7.2% completed an assessment at 4 weeks [23]. The study of Intellicare apps described those who used each app 10 or more times as “active users.” This group comprised 4.7% to 35.7% of users for each app [30].

**Table 1:** Intervention characteristics.

Publication	Intervention	Condition treated	Therapeutic modality	Intervention length	Gamification	Navigation	Evidence from prior trials
Christensen et al (2002) [24] Christensen et al (2004) [25]	MoodGYM Mark I (online program)	Depression/ mood	CBT^a^	5 modules	Includes an interactive game	Sequential	Significant therapeutic effects in an RCT^b^
Christensen et al (2006) [26] Batterham et al (2008) [27] Neil et al (2009) [28]	MoodGYM Mark II (online program)	Depression/ mood	CBT	5 modules	Includes an interactive game	Sequential	Significant therapeutic effects in RCTs
Al-Asadi et al (2014) [18]	Anxiety Online (suite of 5 online programs)	Anxiety	Not specified	12-week program	No apparent gamification^c^	Either	No
Lara et al (2014) [29]	HDep (online program)	Depression/ mood	CBT	7 modules	No apparent gamification^c^	Either	No
Menzies et al (2016) [19]	CBTPsych (online program)	Anxiety (social anxiety for stutterers)	CBT	7 modules	No apparent gamification^c^	Sequential	Therapeutic effects in a small pre-post exploratory trial
Owen et al (2015) [20]	PTSD Coach (app)	Anxiety (PTSD)	CBT	No specified length	No apparent gamification^c^	Open	Pilot RCT: nonsignificant effects Subsequent RCT: significant therapeutic effects
Lattie et al (2016) [30]	Intellicare Apps (suite of apps)	Anxiety and depression	Mixed (includes CBT & positive psychology)	No specified length	Includes some gaming elements in one or more apps	Open	Significant effects in a single-arm trial with coaching provided through the program
Carpenter et al (2016) [23]	Happify (app & online program)	Anxiety and mood/ depression	Mixed (includes CBT & positive psychology)	58 core activities	Iincludes some gaming elements and games	Open	No

^a^CBT: cognitive behavioral therapy.

^b^RCT: randomized controlled trial.

^c^On the basis of the study’s description of the program (ie, as at data collection).

**Table 2:** Uptake and usage data.

Publication	Intervention (data collection period)	Registrations/downloads (time period in months)	Average registrations /downloads per month	At least minimal use	Moderate use	Completion or sustained use
Christensen et al (2002) [24]^a^	MoodGYM Mark I (Apr 2001 to Sep 2001)	2909 (6)	485	51.7% completed at least one depression assessment	16% completed at least two depression assessments	Not stated
Christensen et al (2004) [25]	MoodGYM Mark I (Apr 2001 to Sep 2003)	19,607 (30)	654	62% completed at least one depression assessment	15.6% completed two or more modules	0.5% completed a noncompulsory assessment at beginning of the last module
Christensen et al (2006) [26]	MoodGYM Mark II (Sep 2003 to Oct 2004)	38,791 (14)	2,770	69% completed at least one depression assessment	Less than 7% progressed beyond two modules	Not stated
Batterham et al (2008) [27]^b^	MoodGYM Mark II (Jan 2006 to Apr 2007)	82,159 (16)	5135	37% completed one or more modules	10% completed 2 or more modules	Not stated
Neil et al (2009) [28]	MoodGYM Mark II (Jan 2006 to Nov 2007—adolescents)	7207 (23)	313	40.6% completed one or more module*s*	11.1% completed 2 or more modules	2.8% completed all 5 modules
Al-Asadi et al (2014) [18]	Anxiety Online (Oct 2009 to Jan 2012)	9394 persons completed assessment^c^ (28)	336	33.1% accepted and commenced self-help program^d^	Not stated	3.7% of those who completed the first assessment also completed the post-treatment assessment
Lara et al (2014) [29]	HDep (Mar 2009 to Apr 2013)	17,318 persons registered and entered site at least twice^e^ (50)	346	71.4% completed the first module	10% of users did module 4 (users could miss modules, so may not have completed 4)	Not stated
Menzies et al (2016) [19]	CBTPsych (Aug 2011 to Mar 2014)	267 (32)	8	88% logged on at least once	39% completed 4 or more modules	19.5% completed all 7 modules
Owen et al (2015) [20]	PTSD Coach (Mar 2011 to June 2014)	153,834 (36)	4273	61.1% returned to use the app after the day it was installed	52.1% continued to use app 1 week after installation; 41.6% continued to used app 1 month after installation	No specific completion point; however, 28.6% continued to use the app after 3 months, 19.4% continued after 6 months, and 10.6% after 1 year
Lattie et al (2016) [30]	Intellicare Apps (Sep 2014 to Oct 2015)	5210 (13)	401	84.1% of downloaded apps were launched at least once; between 38.7% and 70.2% of users used apps for at least one day	Between 4.7% and 35.7% (depending on specific app) of active users used the app on 10 or more occasions; between 13.1% and 23.3% used the app for 28 or more days	Not stated
Carpenter et al (2016) [23]	Happify (Dec 2014 to May 2016)	Total downloads not reported. 720,952 persons completed an assessment (18)^f^	40,053	21.2% of those who had completed an assessment at registration completed at least one more assessment^f^	20.6% also completed an assessment at 2 weeks, 7.2% completed an assessment at 4 weeks^f^	3.5% completed an assessment at 6 weeks, 2.1% completed an assessment at 8 weeks

^a^The timeframe covered in this study is a subset of that reported by Christensen et al [25] for the same intervention, but the 2002 study reports some data that are not reported in the 2004 study.

^b^The timeframe reported in this study is a subset of that reported by Neil et al [28] for the same intervention. However, this study reports data for all registrants, whereas Neil et al’s [28] study only reports data for adolescent users.

^c^Persons (n=9394) completed an online assessment and were then offered an online self-help or therapist-assisted program. No other indications of registration are reported.

^d^Persons (n=3107) selected and commenced an online self-help program; they did not formally withdraw and were not recorded as “in progress” at the time of the publication.

^e^No other registration data were reported.

^f^Assessments were not compulsory.

Only 2 studies directly reported intervention completion rates. In these cases, 2.8% of MoodGYM users completed all 5 modules [28] and 19.5% of CBTPsych users completed the program [19]. Looking at other indicators of sustained use for the online programs, 0.5% of MoodGYM Mark I users completed a noncompulsory assessment in the final module [25]. In total, 3.7% of Anxiety Online users who had completed an initial assessment and were offered a self-help online intervention also completed a post-treatment assessment [18]; however, these users may or may not have been utilizing the treatment. There were no specific completion data reported for the apps. However, 19.4% of PTSD Coach users continued to use the app after 6 months [20] and 3.5% of Happify users completed a 6-week assessment [23], although again, these users may have completed assessments without engaging in other content.

Combining completion or sustained use data with the number of people beginning each intervention (uptake), as reported here, suggests that fewer than 40 persons per month completed final assessments or final modules for any of the online modular programs during the study periods. In contrast, over 800 users completed the Happify app assessments at 8 weeks [23], and over 1000 persons per month demonstrated continued use of PTSD Coach after 3 months [20].

## Discussion

### Summary of Evidence

For digital mental health interventions to have a population-level impact, significant numbers of people must receive beneficial doses. This requires both sufficient uptake and ongoing use of effective interventions. Despite over 10,000 digital mental health interventions being publicly available in 2017 [36], we identified only 11 peer-reviewed publications reporting uptake and/or usage data from publicly available digital self-help interventions for depression, anxiety, or low mood. This is disappointing, given 3 considerations. First, people may use interventions differently in the real world, as compared with trial conditions. Second, digital interventions allow relative ease of data collection through automation. Third, comparisons of uptake and usage across interventions could inform improvements in the field. Where data have been reported, diverse measures were used, making direct comparisons challenging. Nevertheless, large differences are apparent. The widest-reaching intervention in our review had tens of thousands of new users per month, whereas the least used one had fewer than ten. Moreover, ongoing use ranged from less than 1% to over 28% of users completing interventions or demonstrating sustained use.

The findings suggest that people may use digital mental health interventions differently in real-world settings, as compared with trial conditions. Although this may be true for many interventions, the phenomenon is easily quantifiable in digital interventions through embedded routine data collection. Completion rates, as reported in included studies, are lower than the completion rates of 43% to 99% in a systematic review of adherence in controlled trials of online interventions for depression and anxiety [7]. Direct comparisons between research trials and implementation usage data for the same intervention also suggest reduced adherence in real-world settings. For example, only 0.5% of community users (ie, users of MoodGYM as publicly implemented and freely available online) of MoodGYM completed a noncompulsory final assessment, compared with 22.5% of participants in a trial evaluating the same program [31]. Similarly, in the community, adolescent users of MoodGYM completed an average of 3.1 exercises, compared with an average of 9.4 exercises among adolescents in a school-based trial [28].

**Recommended reporting of real-world implementation data.**

The following data should be reported from implemented digital mental health interventions:

- total number of registrants or downloads over a specified time period
- the characteristics (such as demographic details) of registrants and users where available
- the number of modules/levels or activities that can be completed and that are completed by users
- number of times the intervention has been accessed and/or the amount of time the user logged on
- the number or percentage of persons completing a “therapeutic dose” of the intervention
- clinical change or effectiveness measures as well as number or percentage of users with clinically significant improvements and deteriorations.

Previous studies have highlighted differences in adherence between guided and unguided self-help interventions [8]. Given the limited, heterogeneous data, we did not conduct meta-analyses or test for differences between interventions. However, on the face of it, a long established program (MoodGYM released in 2001) [24] and 2 of the recently tested app-based interventions (PTSD Coach [20] and Happify [23]) appear particularly promising in terms of uptake. Two interventions reported high sustained use. The first was CBTPsych [19], the 7-module online CBT program for addressing social anxiety among stutterers. Very few people used this, but retention was high. Second, a very different intervention, PTSD coach (available as an app, with no fixed length and an open navigation structure), had high sustained use [20]. Over 10 times as many people engaged in sustained use of apps as completed any of the online modular programs.

An issue for consideration is that of what is a beneficial dose at a population level. The dose or amount of exposure to digital mental health interventions for clinically significant effects has been considered in previous research, with greater adherence generally associated with greater clinical gains [6,7,9]. Included studies show that large numbers of people accessed some mental health interventions for brief periods. Relatively brief use might have a significant population impact if this exposes large numbers of people to ideas such as depression being common, there being a range of ways to address it, and help being available. Future research should consider this.

Perhaps, the strongest implication from this study is that future research should report intervention uptake, ongoing use, and impact in real-world settings. Transparent reporting of key data, such as those as shown in Textbox 1, would facilitate comparisons. Alongside these data, reporting of intervention characteristics, modes of delivery, and features of implementation such as marketing and methods of dissemination would provide opportunities for understanding which interventions, applied in which ways, engage and retain users.

### Strengths and Limitations

This review examined data from peer-reviewed articles. We did not examine grey literature or request data from providers of interventions or Internet service providers due to resource constraints. Further research should explore this. There is a risk of publication bias, given that interventions with poor results may not be reported. A meta-analysis was not conducted due to the small number of published studies and the heterogeneity of data. However, with increased data and more consistent reporting, a meta-analysis would be a valuable future addition to the literature. We set inclusion and exclusion criteria to focus on interventions addressing the very common issues of depression, anxiety, and low mood. We included an intervention targeting PTSD. PTSD was included as an anxiety disorder in the edition of the Diagnostic and Statistical Manual/DSM-IV-TR [21], which was in use at the start of our review period (2002-2017), but not in the fifth edition, issued in 2013 [22]. Others might have made different decisions; however, we have endeavored to be transparent in this. These limitations notwithstanding, this is the first systematic review of implementation data in this area, and it highlights valuable opportunities for development.

### Conclusion

Digital self-help interventions targeting depression, anxiety, or the enhancement of mood have the potential to improve population-level mental health in a highly scalable manner. However, for these interventions to achieve meaningful impact, they need to have adequate uptake and adherence in real-world settings. Only a handful of interventions have reported this information in the peer-reviewed literature to date, and these utilized diverse measures. Nevertheless, the published studies of unguided self-help interventions for anxiety, depression, and mood demonstrate large differences in uptake and engagement between interventions. Organizations delivering these interventions should take advantage of the opportunity to gather and publish data. Much of the data collection on intervention usage can be automated, making such collection and subsequent reporting generally easy and low-cost. We have proposed key metrics that should be considered. Transparent, comparable, and timely publication of real-world data would allow between-program comparisons and hence facilitate improvements in user uptake and engagement, arguably 2 of the major challenges in the digital health world.

## Abbreviations

CBT: cognitive behavioral therapy
JMIR: Journal of Medical Internet Research
RCTs: randomized controlled trials
